# Basal cell carcinoma of the prostate: clinicopathologic analysis of three cases and a review of the literature

**DOI:** 10.1186/1477-7819-11-193

**Published:** 2013-08-13

**Authors:** Kun Chang, Bo Dai, YunYi Kong, YuanYuan Qu, JianNong Wu, DingWei Ye, XuDong Yao, ShiLin Zhang, HaiLiang Zhang, Yao Zhu, WeiQiang Yao

**Affiliations:** 1 Department of Urology, Fudan University Shanghai Cancer Center, 136 Yi Xue Yuan Road, Shanghai 200032, China; 2 Department of Oncology, Fudan University Shanghai Medical College, 136 Yi Xue Yuan Road, Shanghai 200032, China; 3 Department of Pathology, Fudan University Shanghai Cancer Center, 136 Yi Xue Yuan, Shanghai 200032, China; 4 Department of Pathology, Affiliated Hospital of Jiangsu University, Zhenjiang, Jiangsu 212001, China; 5 Department of Radiation Oncology, Fudan University Shanghai Cancer Center, 136 Yi Xue Yuan Road, Shanghai 200032, China

**Keywords:** Basal cell carcinoma, Prostate, Immunochemistry

## Abstract

**Background:**

Although conventional adenocarcinoma accounts for the majority of prostatic carcinomas, it is important to recognize rare variants, like basal cell carcinoma (BCC), which has distinctive histopathological and biological features.

**Case report:**

We analyzed three cases of prostatic BCC and all of them complained of acute urinary retention and digital rectal examination disclosed a stony hard prostate. However, all of them presented with low prostate-specific antigen. The diagnosis relied on transrectal ultrasound-guided needle biopsies or transurethral resection of the prostate (TURP). The microscopic findings suggested basaloid cells with large pleomorphic nuclei and scant cytoplasm, showing peripheral palisading and forming solid nests, and immunohistochemical markers like 34βE12, p63 and Ki67 staining, were positive. After active treatment, two of the patients are alive with tumor and one died five months after discharge from our hospital.

## Background

Basal cell carcinoma (BCC) of the prostate is extremely rare among prostatic carcinomas, and few cases have been reported in the literature. In 2007, Ali and colleagues reported 29 cases of BCC of the prostate [1], which account for the largest case series so far. Most other related published papers are case reports and each of them only reported one case, so further research is desperately needed. The objectives of our study were to retrospectively analyze the three cases treated in our department, elucidate the clinical and pathologic features and discuss the therapeutic strategies.

## Case presentation

### Case 1

**Figure 1:**
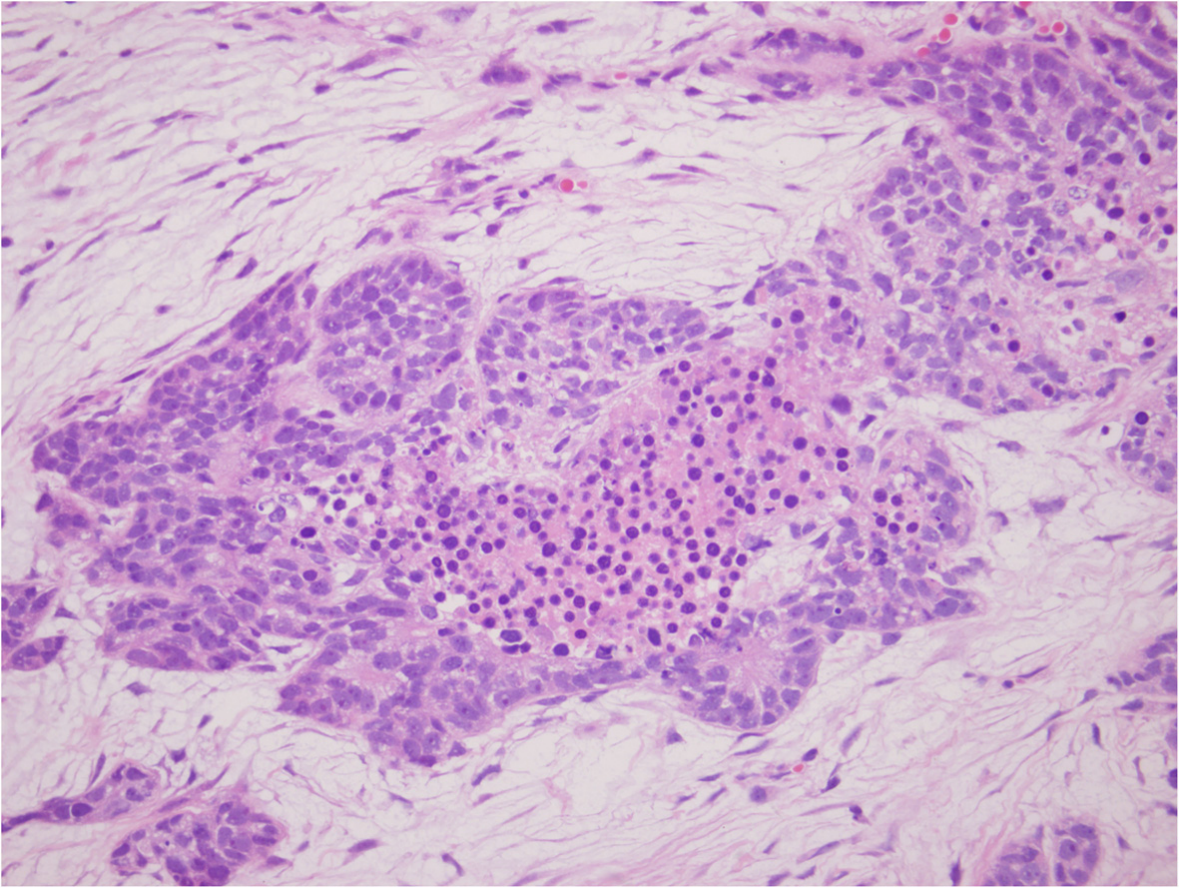
**Histopathology of the resected specimen.** Basaloid cells with large pleomorphic nuclei and scant cytoplasm, showing peripheral palisading and forming solid nests.

**Figure 2:**
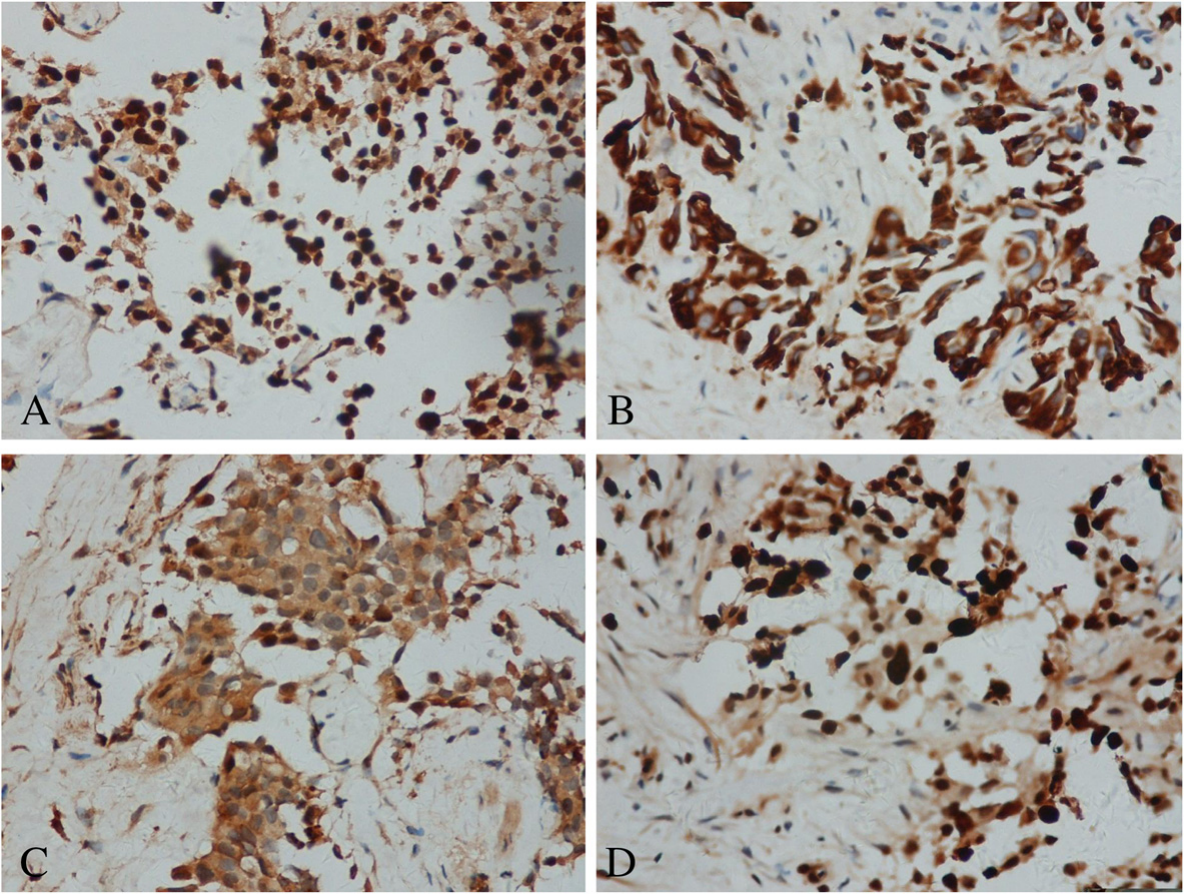
**Immunohistochemical findings in the prostate tumor (A-D). (A)** Basal cell carcinoma: p63 immunohistochemical expression (original magnification ×400); **(B)** basal cell carcinoma: 34βE12 immunohistochemical expression (original magnification ×400); **(C)** basal cell carcinoma: bcl-2 immunohistochemical expression (original magnification ×400); **(D) **basal cell carcinoma:the fraction of Ki-67-positive cells among tumor cells is approximately 70% (original magnification ×400).

**Figure 3:**
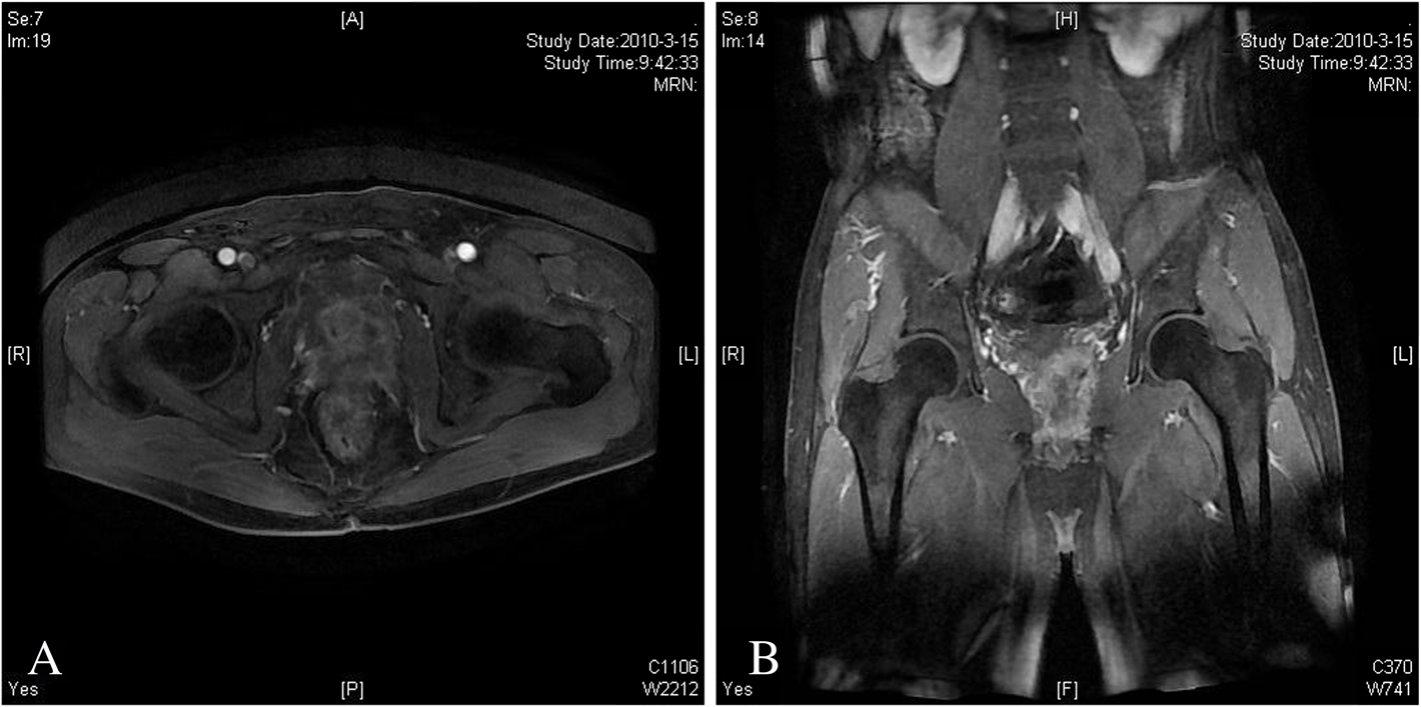
Magnetic resonance imaging: Both the axial (A) and the coronal (B) images suggested extra prostatic extension and the invasion of the bladder wall and the rectum.

**Figure 4:**
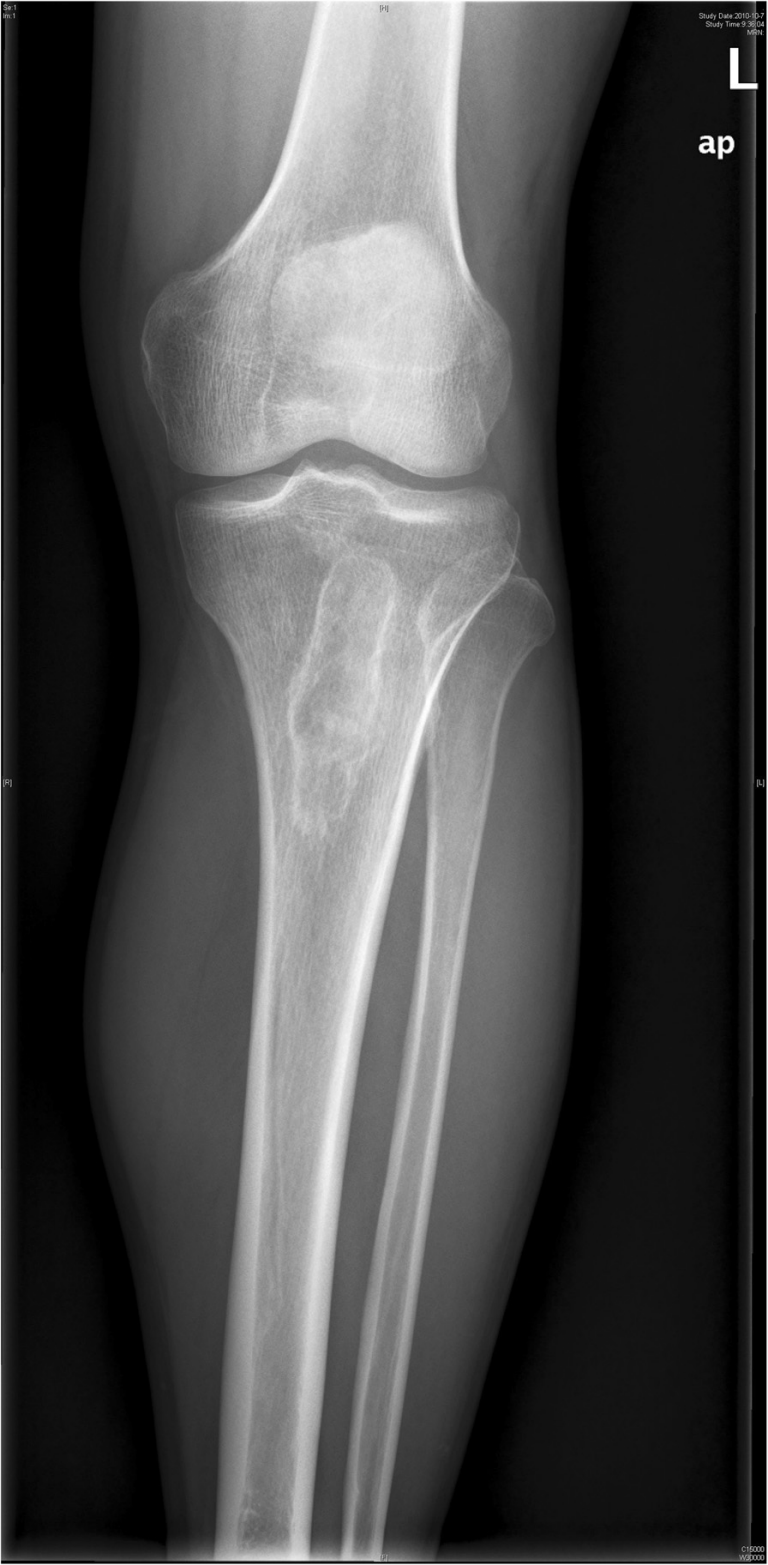
Metastasis at the superior tibia on the left leg.

A 48-year-old man with a five-year history of difficult urination was admitted to the local hospital due to acute urinary retention. A digital rectal examination (DRE) disclosed an enlarged, hard, fixed nodular prostate on the left lobe. However, his serum prostate-specific antigen (PSA) was within normal range (0.29 ng/mL). A prostate biopsy was performed and microscopic examination revealed a malignant tumor composed of basaloid cells with large pleomorphic nuclei and scant cytoplasm, showing peripheral palisading and forming solid nests (Figure 1), with extensive infiltration among benign prostatic glandular elements. Immunoreactivity of the tumor for 34βE12, bcl-2, p63, p53, and Ki67 (Figure 2A, B, C, D), coupled with the absence of negative staining for PSA and cytokeratins 7 and 20, strongly favored a diagnosis of BCC [2,3]. However, a magnetic resonance imaging (MRI) scan reported extra prostatic extension (EPE) and the invasion of the bladder wall and the rectum (Figure 3). The patient was then transferred to our hospital. Considering the health condition of the patient and that no evidence of distant metastasis was found, we decided to treat the patient with three-dimensional conformal radiation therapy with a total dosage of 50Gy in 25 fractions. After two months of treatment, MRI suggested that the primary lesions were significantly reduced with no evidence of metastasis. Therefore, a radical cystectomy with ileal conduit urinary diversion was performed. Hormonal therapy was initiated with a luteinizing hormone-releasing hormone agonist, goserelin (Zoladex), and an antiandrogen agent, flutamide. However, four months later, the patient complained of bone pain and a whole body bone scan documented metastases at the lower part of the ilium, sacroiliac joint and the superior tibia on his left leg (Figure 4). After multidisciplinary consultation, we decided to treat the patient with 30.0Gy of palliative radiotherapy delivered in 10 fractions to the painful osseous metastases plus zoledronic acid. Six months later, multiple hepatic metastases were documented by MRI, among which the biggest one turned out to be around 1.5 cm in diameter. We replaced hormonal therapy with docetaxel as chemotherapy and the patient received hepatic intervention therapy simultaneously. We have followed up this patient for nearly three years and now the patient can perform daily activities independently and has an Eastern Cooperative Oncology Group (ECOG) score of 1 (Table 1).

**Table 1:** The characteristics of our patients and the therapeutic strategies were summarized

**Patient**	**Age(years)**	**Presentation**	**EPE**	**Ki67%**	**Regional treatment**	**Hormonal therapy**	**Metastases**	**Outcome**	**Regional treatment to metastasis, month**	**Presentation to outcome, month**
1	50	Obstruction	+	70	Radical cystectomy + ileal conduit urinary diversion	Yes	Bone, Liver	Alive with tumor	4	58
2	62	Obstruction	+	50	TURP + radiation therapy	Yes	Bone	Alive with tumor	12	38
3	65	Obstruction	+	60	TURP	Yes	Lung	Died of tumor	2	5

*EPE*, extraprostatic extention; *TURP*, transurethral resection of the prostate.

### Case 2

A 62-year-old man received transurethral resection of the prostate (TURP)in the local hospital due to acute urinary retention and his PSA was 1.36ng/ml before the operation. A pathological evaluation established a diagnosis of basal cell carcinoma and it was confirmed by immunohistochemical results, staining positive for 34βE12, bcl-2, p63 and Ki67. DRE also revealed that the invasion of the rectum and surgery may include stool diversion. As a result, radical radiation therapy was performed and the treatment effect was satisfactory in that the MRI showed shrinkage of the tumor volume. Hormonal therapy followed the radiation therapy. A bone scan reported an osseous metastasis on the right side of the is chium 30 months after radical radiation therapy and a palliative radiotherapy followed. Now, the patient’s condition is deteriorating rapidly and he has an ECOG of 3 (Table 1).

### Case 3

A 65-year-old man underwent TURP in the local hospital for acute urinary retention and his serum PSA was 1.35 ng/ml. After the slides consultation in our hospital, BCC was diagnosed as the tumor cells strongly expressed p63, Ki67, 34βE12 and bcl-2. Considering the bilateral pulmonary metastasis and the patient’s poor health condition, only hormonal therapy with goserelin (Zoladex) plus bicalutamide (Casodex) was performed. The patient died five months after discharge from our hospital (Table 1).

## Discussion

The age of patients with BCC of the prostate ranges from young to older individuals (28 to78 years; mean, 50 years) with variable symptoms including nocturia, urgency, or acute urinary retention [3]. The enlarged and partly indurated prostate gland on rectal examination is an important finding toward a clinical diagnosis [4].

Although prostate adenocarcinoma is always monitored by elevated PSA level, the serum PSA of BCC is usually within the normal range. Almost all the prostatic BCC with preoperative elevated PSA are diagnosed with concurrent prostate adenocarcinoma (Table 2). This may be explained by its assumed histological origin: the basal cells, which have no secretory function [5]. Lacking typical clinical manifestations, the diagnosis relied on the pathological findings and the immunohistochemistry results.

**Table 2:** Serum prostate-specific antigen (PSA) level in prostate basal cell carcinoma(BCC)

**Author**	**Cases**	**Pathological diagnosis**	**Preoperative elevated PSA cases (%)**
Iczkowski *et al*. [3]	15	BCC	1 (6.7%)
	4	BCC+acinar carcinoma	4 (100%)
Ali* et al*. [1]	28	BCC	0 (0%)
	4	BCC+acinar carcinoma	1 (25%)
Our cases	3	BCC	0 (0%)

According to the different pathological morphology, BCC can be histologically divided into adenoid-cystic and basaloid variants. Basaloid pattern is always infiltrated by irregular solid clumps, trabeculae and larger cellular masses composed of basaloid cells [6]. The cells have uniform large pleomorphic nuclei and scant cytoplasm. While there is peripheral palisading and forming of solid nests, cribriforming is absent or minimal. The adenoid-cystic type is characterized by cylinders of hyalinized or mucinous stroma surrounded by nests of small epithelial cells, which give the tissue a perforated, sieve-like or cribriform appearance [4]. All of our cases turned out to be the basaloid type. On immunohistochemistry, BCC tumor cells usually show a variable degree of positive immunostaining for p63 and high-molecular-weight cytokeratin 34bE12, both of which are suggestive of BCC. Conventional adenocarcinoma of the prostate is negative for these antibodies [7].

For this rare malignancy, the differential diagnosis is indispensable, including benign basal cell hyperplasia (BCH), poorly differentiated adenocarcinoma and poorly differentiated squamous carcinoma. The differences between benign hyperplasia and the malignant can be drawn from the invasive growth pattern, extensive infiltration, perineural invasion, EPE, and the presence of necrosis. An elevated Ki67 labeling index can also allow distinction between benign and malignant prostatic basal cell lesions. Poorly differentiated adenocarcinoma may be nonreactive for PSA, which turned out to be hard to differentiate, but the presence of immunoreactivity for cytokeratin 14 and negative staining for high-molecular-weight cytokeratin were helpful in distinguishing it from BCC. Though primary squamous carcinoma may also originate from prostatic urethral epithelial cells, transitional epithelial cells around the urethra, basal cells of the prostatic acini or pluripotent stem cells from the prostate so that some immunohistochemistry marks may be the same as BCC, it has its own characteristics, including epithelial keratinization, obvious intercellular bridges, and lack of acinar structures. These characteristics can be helpful in distinguishing it from BCC.

BCC outcome is currently considered uncertain. McKenney *et al*. suggested that many of the reported BCCs have been diagnosed with no definitive histological evidence of malignancy or aggressive clinical course [8]. Grignon also admitted these lesions as a neoplasm of low malignant potential [9]. However, our presented cases showed malignant behavior with EPE and distant metastasis were found just six months after the regional treatment on average. According to the literature, relatively high Ki67 staining may suggest aggressive behavior. Bohn reported that Ki67 is a useful marker determining proliferation; the proliferative rate of BCH and florid BCH is usually lower than BCC [10]. Among Ali’s reported 29 cases [1], seven of the cases presented Ki67 staining over 25%, and three (42.85%) of them were found with distant metastasis in the follow-up. While there were only four cases (13.79%) found with distant metastasis in all. Combined with our cases, which all presented Ki67>25%, we are inclined to draw the conclusion that relatively high Ki67 staining was related to the distant metastasis. However, more cases are needed to analyze whether there is a correlation between high Ki67 staining and BCC distant metastasis. Therefore, it alarms us that aggressive treatment is required as soon as the diagnosis of BCC is established.

There is no consensus on treatment for prostatic BCC. The commonly accepted effective treatment is radical retropubic prostatectomy (RRP) [5]. Though there are few reported treating experiences about RRP, it was helpful in shrinking the size of the primary tumor volume in our two cases. But its effect on preventing the tumor cells from metastasis may be limited, as distant metastases were found in the following few months.

It is still controversial whether hormonal therapy is effective in treating prostatic BCC. Koochekpour reported that androgen suppression therapy is the gold standard, first-line therapy for advanced/metastatic disease [11]. However, Segawa reported that their patient attempted androgendeprivation therapy, but without success [12]. All of our three cases also received hormonal therapy but no evidence was found that it was helpful with their prostatic BCC. Combined with BCC’s origin, which is stated above, we are inclined to conclude that hormonal therapy has no effect on prostatic BCC. Further study with a larger cohort is needed in the future.

Though distant metastases were found just a few months after the initial treatment, the first patient still lives with a relatively high quality of life after distant metastases were recorded two years ago. So positive treatment according to our treating experience is strongly recommended. Chemotherapy may be helpful but the results are not consistent [13].

An exact prognosis of prostatic BCC is not known due to the scarcity of reported cases. Iczkowski *et al*. reported that a five-year metastatic potential ranges from 5 to 10% in T1/T2 tumor to 50 to85% in stage T3/T4 tumor [14]. This conclusion is partially confirmed by our cases, all of them can be diagnosed as T4 stage and distant metastases were detected within five years after diagnosis.

## Conclusions

Basal cell carcinoma is a rare subtype of prostate cancer. Initial suspicion of malignancy is difficult as the serum PSA level is always within the normal range. The final diagnosis still relies on the pathologic findings and immunohistochemistry markers after TURP or a prostate biopsy. Radical retropubic prostatectomy is still regarded as the most effective way of treating this disease. We also tried radiation therapy, chemical therapy and hormonal therapy, but their effect on BCC needs further investigation. This tumor has a biological potential that allows metastasis in the short term, so close, long-term follow-up is strongly recommended.

## Consent

Written informed consent was obtained from the first two patients and the last patient’s next of kin for publication of this case report and any accompanying images. A copy of the written consent is available for review by the Editor-in-Chief of this journal.

## Abbreviations

BCC: Basal cell carcinoma; BCH: Basal cell hyperplasia; DRE: Digital rectal examination; EPE: Extraprostatic extention; MRI: Magnetic resonance imaging; PSA: Prostate-specific antigen; RRP: Radical retropubic prostatectomy; TURP: Transurethral resection of the prostate.

## Competing interests

The authors declare that they have no competing interests.

## Authors’ contributions

KC and BD conceived of the concept, participated in drafting the manuscriptand conducted the critical review. YYQ collected patients’ partial clinical data and revised the manuscript. YYK and JNW reviewed the pathological slides and revised the manuscript. DWY supervised the project and revised the manuscript. XDY, SLZ, HLZ, YZ and WQY took part in the treatment of the patients, assembled data, and followed up with patients. All the authors read and approved the final version and agreed to publish the manuscript.
